# Imaging the distribution of an antibody-drug conjugate constituent targeting mesothelin with ^89^Zr and IRDye 800CW in mice bearing human pancreatic tumor xenografts

**DOI:** 10.18632/oncotarget.5877

**Published:** 2015-10-29

**Authors:** Eva J. ter Weele, Anton G.T. Terwisscha van Scheltinga, Jos G.W. Kosterink, Linda Pot, Silke R. Vedelaar, Laetitia E. Lamberts, Simon P. Williams, Marjolijn N. Lub-de Hooge, Elisabeth G.E. de Vries

**Affiliations:** ^1^ Department of Clinical Pharmacy and Pharmacology, University of Groningen, University Medical Center Groningen, Groningen, Netherlands; ^2^ Department of Medical Oncology, University of Groningen, University Medical Center Groningen, Groningen, Netherlands; ^3^ Department of Nuclear Medicine and Molecular Imaging, University of Groningen, University Medical Center Groningen, Groningen, Netherlands; ^4^ Department of Pharmacy, Section of Pharmacotherapy and Pharmaceutical Care, University of Groningen, Groningen, Netherlands; ^5^ Department of Biomedical Imaging, Genentech, Inc., South San Francisco, CA, USA

**Keywords:** mesothelin, pancreatic cancer, zirconium-89, PET imaging, IRDye 800CW

## Abstract

Mesothelin is a tumor differentiation antigen expressed by epithelial tumors, including pancreatic cancer. Currently, mesothelin is being targeted with an antibody-drug conjugate (ADC) consisting of a mesothelin-specific antibody coupled to a highly potent chemotherapeutic drug. Considering the toxicity of the ADC and reduced accessibility of pancreatic tumors, non-invasive imaging could provide necessary information. We therefore developed a zirconium-89 (^89^Zr) labeled anti-mesothelin antibody (^89^Zr-AMA) to study its biodistribution in human pancreatic tumor bearing mice. Biodistribution and dose-finding of ^89^Zr-AMA were studied 144 h after tracer injection in mice with subcutaneously xenografted HPAC. MicroPET imaging was performed 24, 72 and 144 h after tracer injection in mice bearing HPAC or Capan-2. Tumor uptake and organ distribution of ^89^Zr-AMA were compared with nonspecific ^111^In-IgG. Biodistribution analyses revealed a dose-dependent ^89^Zr-AMA tumor uptake. Tumor uptake of ^89^Zr-AMA was higher than ^111^In-IgG using the lowest tracer dose. MicroPET showed increased tumor uptake over 6 days, whereas activity in blood pool and other tissues decreased. Immunohistochemistry showed that mesothelin was expressed by the HPAC and CAPAN-2 tumors and fluorescence microscopy revealed that AMA-800CW was present in tumor cell cytoplasm. ^89^Zr-AMA tumor uptake is antigen-specific in mesothelin-expressing tumors. ^89^Zr-AMA PET provides non-invasive, real-time information about AMA distribution and tumor targeting.

## INTRODUCTION

Pancreatic cancer is one of the most aggressive tumor entities, with a dismal prognosis. Despite advances in treatment of solid tumors, pancreatic cancer has a 5 year survival rate of only 6% and a median survival of about 6 months [1]. This poor prognosis is due to its advanced stage at diagnosis and limited curative treatment options.

Until now chemotherapy and targeted agents have yielded only modest effects [2]. This has stimulated a search for new approaches; one such approach is the antibody-drug conjugate (ADC), which consists of a specific antibody armed with a highly potent chemotherapeutic drug that will be released after internalization. Mesothelin might serve as a target for ADCs. It is a tumor differentiation antigen highly expressed by cells of many epithelial cancers, with limited expression in normal human tissues [3]. Mesothelin is overexpressed in mesothelioma, ovarian cancers, and in 70–100% of pancreatic adenocarcinomas [4, 5]. Interestingly, its expression in normal tissues is limited to mesothelial cells on pleura, pericardium and peritoneum [6, 7]. A number of early clinical results suggest the validity of using mesothelin as target. In a phase II study with the anti-mesothelin immunotoxin SS1P combined with pentostatin and cyclophosphamide, three out of 10 evaluable patients showed major regression [8]. In addition, in a phase I study combining SS1P with pemetrexed and cisplatin 12 out of 20 patients had a partial response and three showed stable disease [9].

The precise dosing of drugs and selection of patients for mesothelin directed therapy could benefit from non-invasive visualization of anti-mesothelin antibody (AMA) to quantify antibody uptake in tumor lesions, and ultimately to relate this to anti-tumor effects of mesothelin-targeting ADC. Labeling AMA with long-lived positron zirconium-89 (^89^Zr) might allow non-invasive tracking using PET. ^89^Zr-labeled antibodies have already been studied in preclinical and clinical setting [10, 11].

The aim of this study was to develop and evaluate ^89^Zr-labeled AMA for non-invasive tracking of biodistribution towards pancreatic tumors and organs of interest in mice. In addition, a near infrared (NIR) fluorescent dye (IRDye 800CW) was labeled to AMA to investigate tracer distribution at cellular level.

## RESULTS

### Conjugation, ^89^Zr labeling and quality control of AMA

Size-exclusion high-performance liquid chromatography (SE-HPLC) analysis indicated a good conjugation of AMA to the chelator tetrafluorphenol *N*-succinyldesferal (TFP-*N*-sucDf, VU Medical Center). The reaction started with desferal-antibody ratios (DfAR) of 2:1 and 5:1 and resulted in an effective conjugation of 69% and 65% respectively, and therefore a true DfAR of 1.3:1 and 3.5:1. Quality control also showed less than 5% aggregates of the conjugated antibody. *N*-suc-Df-TFP-AMA was labeled with ^89^Zr; the resulting ^89^Zr-*N*-sucDf-AMA is hereafter referred to as ^89^Zr-AMA. Labeling resulted in a high radiochemical efficiency of 93.4% ± 2.3% (*n* = 5), without further purification, and a high specific activity (>500 MBq/mg). The 1.3:1 DfAR obtained a maximum specific activity of 200 MBq/mg, however, this is insufficient to label the amount of radiation needed for microPET scans for all the AMA doses of interest. Therefore the 3.5:1 DfAR was used in further experiments.

^89^Zr-AMA was radiochemically stable in solution (0.9% NaCl) when stored at 4 and 20°C for over 168 h. Protein-bound ^89^Zr decreased minimally; from 98.3% to 98.0% after storing it for 7 days at 4°C, and from 98.3% to 96.4% after 7 days at 20°C (Supplementary Figure 1A).

**Figure 1:**
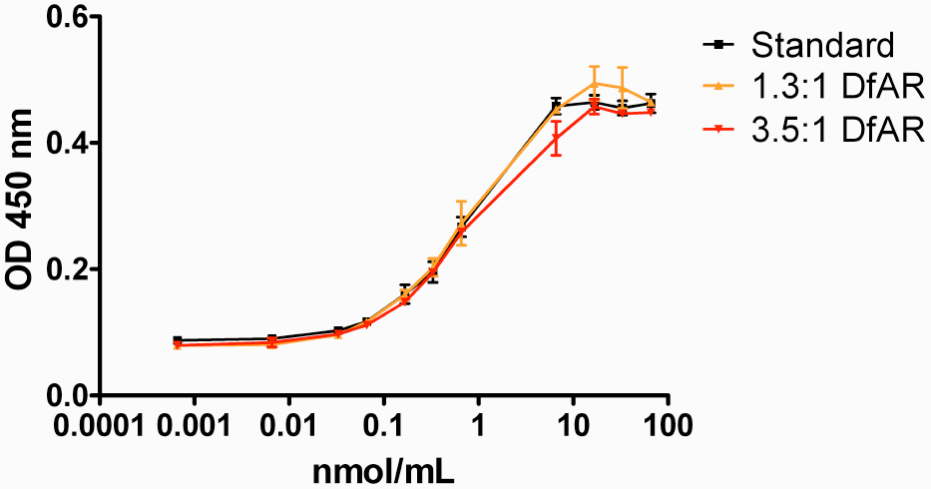
ELISA assay of binding affinity for mesothelin extra cellular domain with AMA conjugated to chelator, ratio 1:1.3 (yellow) and ratio 1:3.5 (red) compared to control (AMA, black) *N* = 3 for each ratio. X-axis depicts the amount of antibody added in nmol/mL; the Y-axis represents the optical density of the fluorescent signal at 450 nm.

DfAR conjugation in ratios of 1.3:1 or 3.5:1 did not affect binding affinity of AMA (*P* < 0.05, Figure 1). Immunoreactivity assay of ^89^Zr-AMA showed ~50% inhibition of the maximum binding of 14 nM AMA for competition of extracellular domain of mesothelin binding of 14 nM ^89^Zr-AMA, indicating a fully preserved immunoreactivity.

### Dose-escalation and biodistribution studies

**Figure 2:**
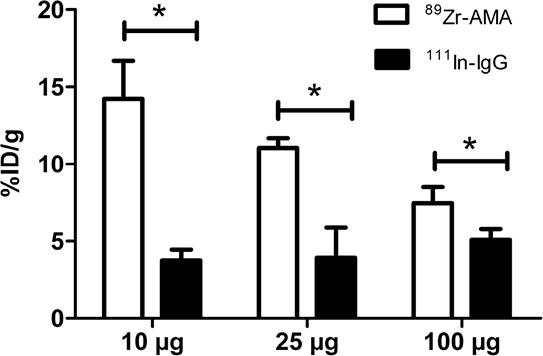
Tumor uptake of 10, 25 and 100 μg of ^89^Zr-AMA (white bars), compared to a same dose of co-injected non-specific ^111^In-IgG (black bars) *N* = 6 for each dose. The X-axis indicates the doses tested; the Y-axis indicates the percentage of the injected dose that accumulated in tumor corrected for tumor weight in grams. *P < 0.05.

Biodistribution studies in mice with HPAC tumors showed specific tumor uptake of ^89^Zr-AMA compared to nonspecific control for all three doses of 10, 25, and 100 μg (*P* < 0.05, Figure 2). Nonspecific IgG was labeled with ^111^In in order to be able to distinguish between nonspecific uptake and specific ^89^Zr-AMA uptake in the same mouse. This co-injection of tracers allows correcting for potential inter-individual differences. At 144 h after injection, the highest percentage tumor uptake was seen in the 10 μg dose group which was almost 4 times higher than nonspecific control (14.2% ID/g ^89^Zr-AMA vs. 3.7%ID/g ^111^In-IgG; *P* < 0.05, Figure 2 and Supplementary Table 1). Tumor uptake decreased with increasing doses of AMA (*P* < 0.05, one way analysis of variance) from 14.2 ± 2.5%ID/g with 10 μg dose, to 11.1 ± 0.6%ID/g with 25 μg dose, and 7.5 ± 1.1%ID/g with 100 μg dose (Figure 2). *Ex vivo* analysis of isolated organs indicated a normal distribution of ^89^Zr-AMA and ^111^In-IgG. Both tracers showed a similar uptake pattern in most organs in all groups of mice, with few exceptions. ^89^Zr-AMA tumor uptake was higher than ^111^In-IgG with every dose (respectively 3.8, 2.8, and 1.5 fold higher), indicating tumor specific uptake. Bone also showed a 3.5 fold higher activity for ^89^Zr-AMA than nonspecific control. At 10 μg ^89^Zr-AMA tumor-to-blood ratio was 3.08 ± 0.55 and tumor-to-muscle ratio 15.57 ± 5.61. With increasing doses these ratios decreased, indicating dose dependent and saturable tracer distribution.

### MicroPET and IVIS imaging

**Figure 3:**
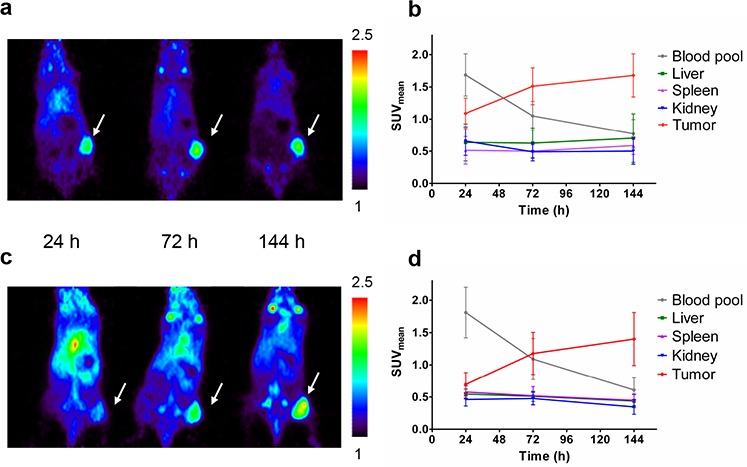
MicroPET imaging data from tumor-bearing mice injected with 10 μg ^89^Zr-AMA, 5 MBq (*n* = 6) Representative coronal imaging results of one mouse 24 h after injection (left), 72 h (middle) and 144 h (right) bearing **A.** a HPAC tumor or **B.** Quantification of activity uptake (SUV_mean_) in tumor and organs of interest over time in HPAC-bearing mice **C.** a Capan-2 tumor, tumors indicated by arrow, color scale depicting SUV_mean_ values from 1 to 2.5. **D.** or Capan-2-bearing mice.

Based on results from the dose-escalation biodistribution study 10 μg ^89^Zr-AMA was used for imaging experiments. MicroPET scans showed homogeneous ^89^Zr-AMA tracer uptake within the tumors at each time point. Tumor uptake increased visibly over time, whereas activity in blood pool decreased (Figure 3A, 3C).

**Figure 4:**
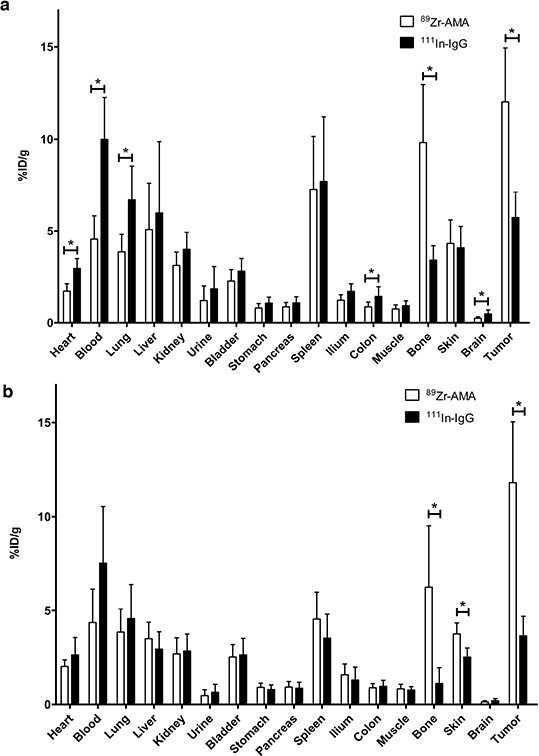
Activity of individual organs containing ^89^Zr-AMA (white bars) and co-injected non-specific ^111^In-IgG (black bars), indicating specific tumor uptake of ^89^Zr-AMA in HPAC tumors (a) and Capan-2 tumors (b) *P < 0.05

Tumor accumulation over time was demonstrated by the mean of standardized uptake value (SUV_mean_) quantification (Figure 3B, 3D). For HPAC tumors it increased from 1.09 ± 0.24, to 1.51 ± 0.29, and 1.68 ± 0.33 from 24 h to 72 and 144 h respectively. For Capan-2 tumors uptake increased from 0.70 ± 0.18, to 1.18 ± 0.32, and 1.40 ± 0.41 at 24, 72, and 144 h after tracer injection. *Ex vivo* ^89^Zr-AMA uptake proved to be tumor-specific in both cell lines (Figure 4A, 4B and Online Resource, Supplementary Table 1), and tumor uptake was consistent with imaging data as %ID/g correlated with SUV_mean_ (see Supplementary Figure 2). ^89^Zr-AMA tumor uptake was similar for the HPAC and Capan-2 xenografts (*P* = 1.000), and quantification of tumor, liver, spleen, and kidney showed comparable results for both cell lines.

**Figure 5:**
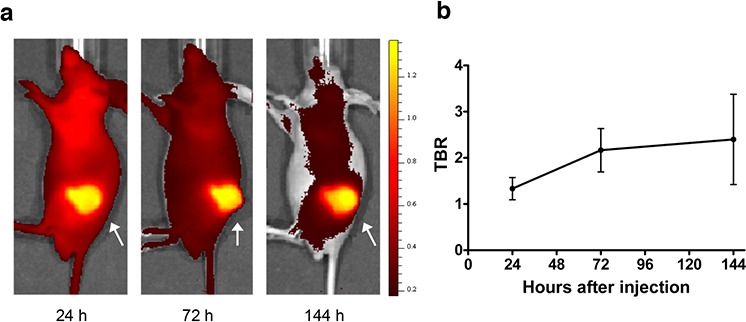
Fluorescence imaging data from HPAC-bearing mice injected with 100 μg AMA-800CW (*n* = 10) **A.** Representative sagittal fluorescence imaging results of one mouse 24 h after tracer injection (left), 72 h (middle) and 144 h (right). The tumor is indicated by an arrow. **B.** Relative tumor uptake in tumor to background ratio (TBR) over time.

Binding affinity of the AMA molecule for mesothelin ECD was preserved after labeling with 800CW. Of the two ratios (Dye:mAb) tested, the 2:1 label ratio showed no aggregation over time, where the 4:1 ratio did show visible precipitation of labeled protein. Whole body fluorescence imaging of mice showed substantial uptake of AMA-800CW in tumor (Figure 5A) and accumulation over time (Figure 5B).

### FACS analyses cell lines, histology and immunohistochemistry tumors

Mesothelin expression was observed in both HPAC and Capan-2 cells and not in H441 control (data not shown). Mesothelin expression was less pronounced in HPAC cells than Capan-2.

**Figure 6:**
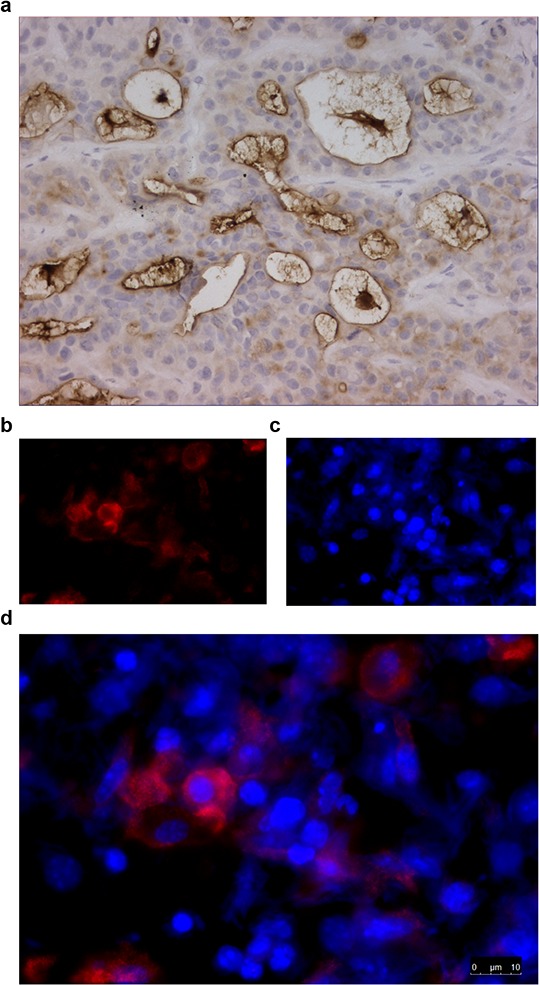
Immunohistochemical staining for mesothelin of HPAC tumor material from mice injected with 100 μg AMA-800CW (tumor harvested 144 h after injection) **A.** A representative brightfield image (400x) of a tumor with an IHC staining for mesothelin. **B.** Fluorescence microscopy at 800 nm for IRDye 800CW labelled to AMA; **C.** at 460 nm for DAPI staining, **D.** and overlay of fluorescent images.

Hematoxylin-eosin (H&E) staining of tumors showed that they consisted purely of tumor tissue and that Capan-2 tumors are more differentiated than HPAC tumors (data not shown). All tumors contained viable tumor cells as well as areas of necrosis due to duct formation, typical for pancreatic tissue. Mesothelin staining was stronger in Capan-2 tumors than in HPAC tumors. Mesothelin staining was present in tumor nests and most strongly expressed at the apical side of tumor cells surrounding ducts (Figure 6A). Fluorescence microscopy revealed that 800CW is mainly located in the cytoplasm of tumor cells (Figure 6B and 6D).

## DISCUSSION

This study shows that it is possible to visualize subcutaneously implanted human pancreatic tumors in mice noninvasively over time with the PET tracer ^89^Zr-AMA. The results indicate a specific mesothelin-driven tumor uptake of targeted ^89^Zr-labeled antibody which visualizes human mesothelin expressing pancreatic adenocarcinoma in real time.

The biodistribution study showed an increase in tumor uptake over time; on average an ^89^Zr-AMA tumor uptake of 14.2% ID/g was seen at 144 h after tracer injection. This may seem a rather low uptake, but a recent study using an ^111^In-labeled anti-ERC/mesothelin mouse monoclonal IgG_2a_ did not exceed 5.8%ID/g in the tumor at any time point of 24, 48 or 96 h [12]. The differences between these studies are likely the consequence of differences in tumor exposure given differences in pharmacokinetics (PK) of the antibodies. Liver showed a relatively high activity with ^111^In-labeled antibody compared with tumor (respectively 15 versus 5.8%ID/g at 96 h), indicating clearance, whereas our ^89^Zr-AMA showed better PK in relation to tumor specificity and blood accumulation, and lower non-specific clearance. This may be explained by the relatively low and controlled conjugation ratio of TFP-*N*-sucDesferal to AMA, minimizing the possible alterations in PK properties of the antibody.

We could accurately visualize tracer tumor uptake in two models of xenografted pancreatic adenocarcinoma tumors with ^89^Zr-AMA PET. ^89^Zr-AMA PET showed specific binding to the target with an increase in radioactivity in tumor over time, while the amount of radioactivity decreased in normal organs. Both xenograft types showed similar results despite the different mesothelin expression. This may be due to the fact that the tracer dose was below the saturation limit of the target, or the fact that different internalization rates might cancel out different cell-surface levels. Tumor-uptake of the tracer exceeded the uptake in surrounding tissue, resulting in a good tumor-to-background ratio. Uptake of control did not exceed uptake in surrounding tissues, making it possible to differentiate between specific and non-specific uptake.

Mesothelin was expressed on cell membrane as indicated by immunohistochemistry (IHC) data. Fluorescent microscopy of AMA-800CW suggests that the tracer is internalized, although target expression on membrane could not be visualized as the Hoechst staining washed away any of the membrane-bound AMA-800CW.

MicroPET data indicated a relatively high activity in joints of the mice of 8.5% with ^89^Zr-AMA but not with ^111^In-IgG, indicating an ^89^Zr-specific mechanism. Bone uptake of ^89^Zr might be due to trans-chelation of ^89^Zr from AMA to plasma proteins or AMA catabolites, and from there deposition in bone [13]. ^89^Zr specifically accumulates in mineralized constituents, like epiphysis [14]. This ^89^Zr-related, antibody non-specific uptake in bone is observed in rodents [15]. However, it has no clinical consequences for imaging in humans, as clinical imaging studies show no visible bone uptake on PET scans [16].

Radiolabeling the naked antibody of an ADC can provide interesting information concerning accessibility of the tumor for antibodies and availability of the target in tumors. Using ^89^Zr-PET to better understand the properties of a therapeutic antibody has been described previously [17]. This is especially important for ADCs, as they are not only dependent on the accessibility of the tumor, but also on the extent and internalization of target present. A trial is analyzing whether an ^89^Zr-trastuzumab PET imaging can predict which patients will not respond to the ADC trastuzumab-DM1. A preliminary report indicated that a combination of pre-treatment, ^89^Zr-trastuzumab PET imaging, and early 2[18F]fluoro-2-deoxy-D-glucose positron emission tomography–computed tomography (FDG-PET/CT), with response assessment after 1 cycle seems potentially capable to identify patients unlikely to show a metabolic response after 3 cycles of T-DM1 [18].

Studying mesothelin with molecular imaging in the clinic may be increasingly of interest giving the increment of treatment options tested that use mesothelin as a target and the broad range of tumor that can express mesothelin [19].

Mesothelin was targeted with a SPECT tracer namely an ^111^In-labeled chimeric antibody. In all patients a tumor-to-background ratio (TBR) of at least 1.2 was observed [20]. Our findings with ^89^Zr-AMA were also translated to a clinical trial. This tracer should potentially make it easier to quantify tracer uptake than the SPECT tracer. We GMP-produced ^89^Zr-AMA for a phase I study of a mesothelin-targeting ADC in pancreatic and ovarian cancer patients [21]. In the main study patients received the ADC study drug comprising this AMA armed with the anti-mitotic drug MMAE. In a side study patients received baseline ^89^Zr-AMA-PET scans before starting ADC treatment. These PET scans were performed to quantify antibody uptake in pancreatic and ovarian tumor lesions and ultimately to relate this uptake to anti-tumor effects (http://ClinicalTrials.gov identifier: NCT01832116).

These findings highlight the ability of ^89^Zr-AMA PET to provide non-invasive, real-time information about AMA distribution and tumor targeting and argue for an expansion of ongoing efforts to use PET imaging in the development of ADCs.

## MATERIALS AND METHODS

### Ethics statement

All applicable international, national and/or institutional guidelines for the care and use of animals were followed.

### Conjugation, ^89^Zr labeling of AMA, and ^111^In labeling of IgG

Anti-mesothelin humanized IgG1 monoclonal antibody was conjugated with TFP-*N*-sucDf, at start DfAR of 2:1 and 5:1. These ratios were chosen based on previous experience [10, 11], a DfAR of 2:1 obtains clear images in the clinic, but a higher specific activity is needed for animal experiments, therefore a DfAR of 5:1 was made. Both DfARs were tested for suitability and labeled with clinical-grade ^89^Zr-oxalate (IBA Molecular) as described previously [22]. ^89^Zr-*N*-sucDf-AMA is hereafter referred to as ^89^Zr-AMA.

*N*-sucDf-AMA and ^89^Zr-AMA were analyzed for conjugation ratios, aggregation and radiochemical purity by SE-HPLC. Storage stability of ^89^Zr-AMA was tested in 0.9% NaCl at 4 and 20°C by determining protein-bound radioactivity using trichloroacetic acid (TCA) precipitation. Before storage the sample was purified by ultra filtration. TCA precipitation was performed in PBS with 0.5% human serum albumin (Sanquin) and 20% TCA. Radioactivity in precipitate and supernatant was determined by a calibrated well-type γ-counter (LKB Instruments).

Immunoreactivity of *N*-sucDf-AMA was tested with an ELISA assay with conjugated and unconjugated AMA. The extracellular domain (ECD) of mesothelin (provided by Genentech) was used to coat a 96-well plate (10 μg/mL, Nunc MaxiSorp, Thermo Fisher Scientific).

Immunoreactivity of radiolabeled antibody was tested as described earlier [10]. The assay was performed with unlabeled and unconjugated AMA and ^89^Zr-AMA using ECD of mesothelin as target. Unlabeled AMA was added in a logarithmic concentration range of 1 ng/mL – 1 mg/mL, while ^89^Zr-AMA was kept at a fixed concentration of 1000 ng/mL.

Human IgG served as a nonspecific control in the *in vivo* experiments. To be able to differentiate between AMA and IgG, ^111^In was chosen as radiolabel for IgG. This allowed co-injection of tracer and control in the same animal, making the animal its own control and reduced the number of animals needed. Human IgG (Sanquin) was conjugated to 2-(4-isothiocyanatobenzyl)-diethylenetriaminepenta-acetic acid (p-SCN-Bn-DTPA) and radiolabeled with ^111^InCl_3_ (Mallinckrodt) on day of use as published earlier [23].

### Cell lines

Human pancreatic tumor cell lines HPAC and Capan-2 (American Type Culture Collection) were cultured in RPMI 1640 medium (Invitrogen) with 10% heat-inactivated fetal calf serum (Bodinco BV) at 37°C in a fully humidified atmosphere containing 5% CO_2_. Short tandem repeat (STR) profiling (BaseClear) affirmed identity. Cell lines were quarantined until screening for microbial contamination and mycoplasma had been performed; these tests were negative. In all experiments mice were inoculated subcutaneously with 5 × 10^6^ HPAC or Capan-2 cells. For analysis of receptor expression, cells were incubated with anti-human mesothelin phycoerythrin (catalog number: FAB32652P, R&D systems). Human lung adenocarcinoma epithelial cell line H441 (American Type Culture Collection) served as negative control. Membrane receptor expression was analyzed using flow cytometry (FACSCalibur, BD Biosciences) with Winlist software 6.0 (Verity Software House).

### Dose escalation and biodistribution studies

Animal experiments were conducted using male athymic mice (BALB/cOlaHsd-*Foxn1*^*nu*^, Harlan). Tumor cell inoculation, tracer injection and microPET imaging were performed using isoflurane in medical air inhalation anesthesia (induction 5%, maintenance 2%). At 6–8 weeks of age the mice were injected subcutaneously in the left flank with HPAC cells in 0.2 mL of 1:1 growth medium and Matrigel (BD Bioscience). Tumor growth was estimated with caliper measurements. Mice were used for experiments when tumors measured at least 100 mm^3^, ~2 weeks after tumor inoculation.

Biodistribution studies were conducted in mice bearing HPAC xenograft tumors to evaluate mesothelin-specific tumor uptake and dose-dependent tumor uptake of AMA. ^89^Zr-AMA and ^111^In-IgG were compared for biodistribution in 3 dose groups: 10, 25, or 100 μg (approximately 0.5, 1.2 and 5.0 mg/kg body weight) of both proteins. Each dose was administered to 6 mice intravenously in the penile vein, with a co-injection of the same dose of nonspecific control, both labeled with ± 1 MBq of respectively ^89^Zr and ^111^In. Animals were sacrificed 144 h after tracer injection and organs were excised, rinsed for residual blood, weighed, and counted for radioactivity. Tissue activity was expressed as percentage injected dose per gram tissue (%ID/g), with an assumption of tissue density of 1 g/cm^3^ [24]. All data were corrected for physical decay using known standards. Subsequently, tumors were split, formalin-fixed and paraffin-embedded, and stored at −80°C for *ex vivo* mesothelin measurements.

### MicroPET and IVIS imaging

Based on dose escalation and biodistribution experiments, a dose of 10 μg was used in animal imaging and distribution studies. For imaging a microPET Focus 220 rodent scanner (CTI Siemens) was used. Two groups of 6 animals were subcutaneously injected with either HPAC or Capan-2 cells.

Approximately 2 weeks after tumor inoculation, 10 μg of AMA labeled with 5 MBq ^89^Zr was injected via the penile vein, with a co-injection of 10 μg of IgG labeled with 1 MBq ^111^In.

Static images of animals were acquired with an acquisition time of 15–60 min at 24, 72 and 144 h after tracer injection. Images were corrected for scatter and attenuation. After image reconstruction, *in vivo* quantification was performed with AMIDE (A Medical Image Data Examiner) software (version 1.0.2; Stanford University) [25] and tumor accumulation was calculated as the mean of standardized uptake value (SUV_mean_). For region-of-interest (ROI) measurements, tumor volumes were manually drawn as described earlier [26], based on tumor weight measured *ex vivo*, assuming a tissue density of 1 g/mL. Animals were sacrificed after the last scan (*t* = 144 h) and organs were excised, rinsed for residual blood weighed and counted for radioactivity. Tumors were split, one part formalin-fixed and paraffin-embedded, and one part stored at −80°C for *ex vivo* measurements.

AMA localization within tumor was studied using fluorescent labeled AMA. For production of the fluorescent tracer 1000 μg AMA was labeled with IRDye 800CW-NHS (LI-COR Biosciences) in a ratio of 2:1 and 4:1 (Dye:mAb) according to manufacturer's protocol. Final product was analyzed as described previously [26]. Immunoreactivity of fluorescent AMA was tested with an ELISA assay. A ratio of 2:1 was used in the animal experiments.

Ten mice were injected subcutaneously with HPAC cells. Approximately 2 weeks after tumor inoculation, when tumors measured at least 200 mm^3^, fluorescent imaging agent (100 μg AMA-800CW) was injected via the penile vein. Fluorescence images using IVIS Spectrum were obtained 24 (*n* = 10), 72 (*n* = 7) and 144 h (*n* = 4) after tracer injection. Following each scan, a number of mice were sacrificed and tumors were excised for *ex vivo* analysis.

Fluorescence images were retrieved by using excitation wavelengths of 745 nm and a filter of 800 nm. Data were analyzed using Living Image 4.3.1 software (Caliper Life Sciences). To determine tumor-to-background ratios (TBR) from NIR fluorescent images, tumor boundary was set as ROI, and the ratio was calculated by comparing tumor uptake with shoulder muscle of the animal as background value.

### *Ex vivo* tissue analysis

Formalin-fixed paraffin-embedded tumors were stained using hematoxylin-eosin (H&E) for histology, and mesothelin expression was immunohistochemically stained with an anti-mesothelin isoform 1 antibody (clone #420404; R&D Systems).

For fluorescence microscopy, tumor slides were stained with Hoechst 33258 (Invitrogen).

### Statistical analysis

Data are presented as mean ± SD. Statistical analysis was performed using the Mann-Whitney test for non-parametric data (Prism, version 5; GraphPad Software). A *P* value of 0.05 was considered significant. The correlation between quantified *in vivo* small-animal PET images and *ex vivo* biodistribution data was estimated by linear regression.

## SUPPLEMENTARY FIGURES AND TABLE


