# Supplementary material for: Imaging the distribution of an antibody-drug conjugate constituent targeting mesothelin with 89Zr and IRDye 800CW in mice bearing human pancreatic tumor xenografts
Source: Oncotarget. 2015 Oct 29;6(39):42081–90. doi: 10.18632/oncotarget.5877 (PMC4747211; doi:10.18632/oncotarget.5877)
Supplement: Supplementary file 1 [file oncotarget-06-42081-s001.pdf]

SUPPLEMENTARY FIGURES AND TABLE

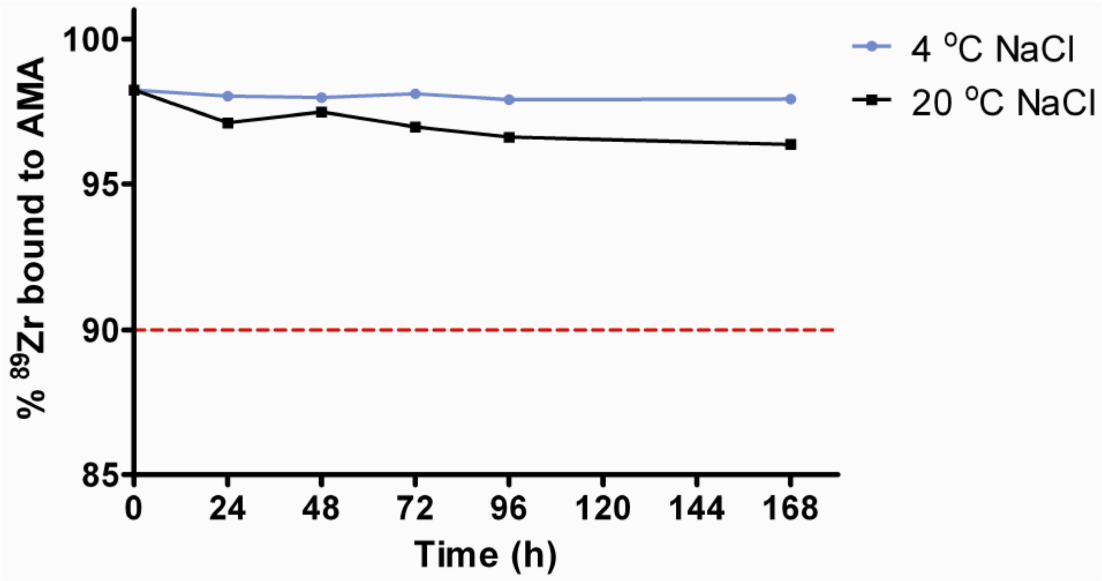

Supplementary Figure S1: Stability testing of ^{89}Zr-AMA. Radiochemical purity was measured at 0, 24, 48, 72, 96, and 168 hours after labeling. A cutoff value of 90% of total ^{89}Zr binding was considered the minimum; any lower would impair *in vivo* scanning and biodistributon, and thus this cutoff was also used to obtain representative *in vitro* data.

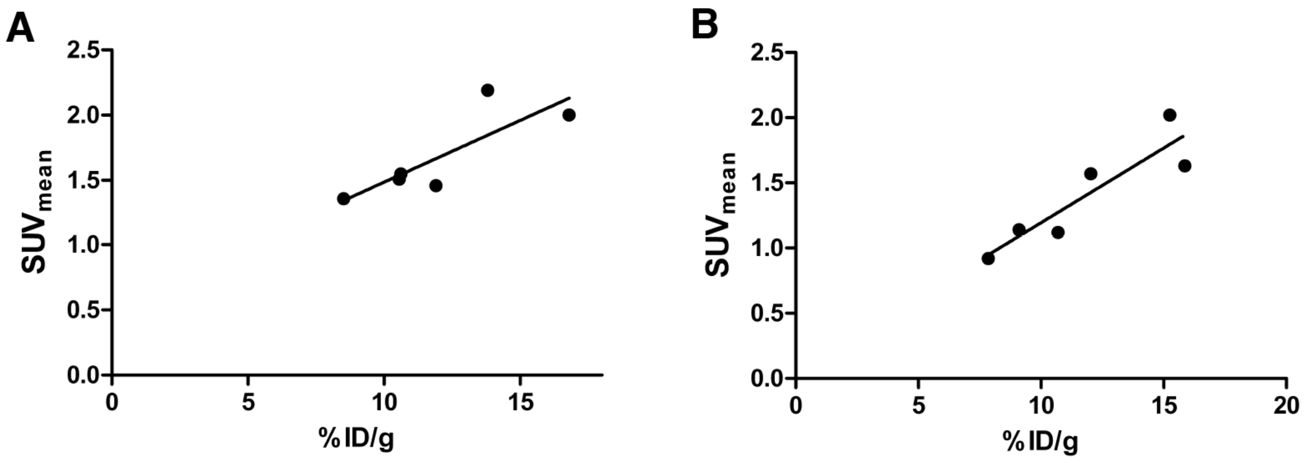

Supplementary Figure S2: Correlation between SUV_{mean} and %ID/g tumor. A. HPAC tumors: $R^2 = 0.68$, $P < 0.05$ (linear regression). **B.** Capan-2 tumors: $R^2 = 0.83$, $P < 0.05$ (linear regression).

Supplementary Table S1: Amount of radioactivity present (^{89}Zr-AMA or ^{111}In-IgG) in organs of interest per injected dose

	Uptake of ^{89}Zr (%ID/g)					Uptake of ^{111}In (%ID/g)				
	Dose escalation			Imaging		Dose escalation			Imaging	
	HPAC			HPAC	CAPAN-2	HPAC			HPAC	CAPAN-2
**Tissue**	**10 µg**	**25 µg**	**100 µg**	**10 µg**	**10 µg**	**10 µg**	**25 µg**	**100 µg**	**10 µg**	**10 µg**
Heart	1.9 ± 0.5	2.1 ± 0.2	1.9 ± 0.2	1.7 ± 0.4	2.0 ± 0.4	2.9 ± 0.9	3.0 ± 0.6	3.0 ± 0.7	3.0 ± 0.5	2.6 ± 0.9
Blood	4.7 ± 0.9	4.7 ± 0.5	3.6 ± 1.3	4.6 ± 1.3	4.4 ± 1.8	11.1 ± 1.7	9.9 ± 1.5	9.6 ± 2.6	10.0 ± 2.3	7.5 ± 3.0
Lung	3.4 ± 0.5	3.6 ± 0.5	2.6 ± 0.7	3.9 ± 0.9	3.9 ± 1.2	5.4 ± 1.5	5.2 ± 0.6	4.8 ± 1.1	6.7 ± 1.8	4.6 ± 1.8
Liver	5.0 ± 1.1	3.8 ± 1.1	3.3 ± 0.6	5.1 ± 2.5	3.5 ± 0.9	4.9 ± 1.1	4.1 ± 1.5	4.0 ± 1.0	6.0 ± 3.9	3.0 ± 0.9
Gallbladder	0.4 ± 0.6	1.1 ± 0.7	1.6 ± 1.8	2.4 ± 1.6	0.9 ± 0.6	1.4 ± 0.9	1.6 ± 1.4	3.1 ± 3.2	3.9 ± 2.4	1.3 ± 1.0
Kidney	3.1 ± 0.4	2.6 ± 0.2	2.1 ± 0.5	3.1 ± 0.7	2.7 ± 0.9	3.9 ± 0.8	3.2 ± 0.7	3.3 ± 0.8	4.0 ± 0.9	2.8 ± 0.9
Urine	0.9 ± 1.4	0.9 ± 0.5	1.1 ± 0.3	1.2 ± 0.8	0.5 ± 0.3	2.3 ± 1.3	1.3 ± 0.9	1.6 ± 0.6	1.9 ± 1.2	0.7 ± 0.4
Bladder	2.2 ± 1.0	2.5 ± 0.5	2.4 ± 0.4	2.3 ± 0.7	2.5 ± 0.7	2.9 ± 1.5	2.8 ± 0.5	3.2 ± 0.5	2.8 ± 0.7	2.6 ± 0.9
Stomach	1.1 ± 0.2	0.9 ± 0.2	0.9 ± 0.1	0.8 ± 0.2	0.9 ± 0.2	1.2 ± 0.3	1.0 ± 0.2	1.1 ± 0.2	1.1 ± 0.3	0.8 ± 0.2
Pancreas	0.9 ± 0.2	1.0 ± 0.1	0.8 ± 0.2	0.9 ± 0.2	0.9 ± 0.3	1.0 ± 0.2	1.0 ± 0.2	0.9 ± 0.2	1.1 ± 0.3	0.9 ± 0.3
Spleen	4.5 ± 1.3	4.2 ± 0.4	3.1 ± 0.6	7.3 ± 2.9	4.5 ± 1.4	5.1 ± 1.8	4.3 ± 0.7	3.7 ± 0.7	7.7 ± 3.5	3.5 ± 1.3
Ilium	1.2 ± 0.4	1.2 ± 0.3	1.1 ± 0.2	1.2 ± 0.3	1.6 ± 0.6	1.4 ± 0.6	1.4 ± 0.3	1.5 ± 0.5	1.7 ± 0.4	1.3 ± 0.7
Colon	1.0 ± 0.2	1.1 ± 0.2	1.0 ± 0.3	0.9 ± 0.3	0.9 ± 0.2	1.4 ± 0.5	1.2 ± 0.2	1.4 ± 0.4	1.4 ± 0.5	1.0 ± 0.3
Muscle	1.1 ± 0.6	0.8 ± 0.2	0.6 ± 0.2	0.8 ± 0.2	0.8 ± 0.2	1.0 ± 0.2	0.9 ± 0.1	0.9 ± 0.2	0.9 ± 0.3	0.8 ± 0.2
Bone	9.5 ± 2.0	10.2 ± 3.0	6.8 ± 3.6	9.8 ± 3.2	6.3 ± 3.3	2.8 ± 1.3	2.9 ± 1.7	1.8 ± 1.3	3.4 ± 0.8	1.1 ± 0.8
Skin	3.9 ± 1.1	3.4 ± 0.4	4.7 ± 1.1	4.3 ± 1.3	3.8 ± 0.6	3.4 ± 0.9	3.1 ± 0.4	3.9 ± 0.5	4.1 ± 1.2	2.5 ± 0.5
Brain	0.2 ± 0.1	0.2 ± 0.1	0.2 ± 0.0	0.2 ± 0.1	0.1 ± 0.1	0.4 ± 0.1	0.5 ± 0.2	0.4 ± 0.1	0.4 ± 0.2	0.2 ± 0.1
Tumor	14.2 ± 2.5	11.1 ± 0.6	7.5 ± 1.1	12.0 ± 2.9	11.8 ± 3.2	3.7 ± 0.7	4.4 ± 1.2	5.1 ± 0.7	5.7 ± 1.4	3.7 ± 1.0

HPAC xenografts used in dose escalation, 10, 25 and 100 µg antibody injected, or imaging study with both HPAC and Capan-2 xenografts, 10 µg antibody injected. Data are presented as mean ± SD.
